# Isolation, Purification and Characterization of L,D-transpeptidase 2 from Mycobacterium tuberculosis

**Published:** 2019

**Authors:** S. M. Baldin, T. A. Shcherbakova, V. K. Švedas

**Affiliations:** Lomonosov Moscow State University, Belozersky Institute of Physicochemical Biology, Leninskie gory 1, bldg. 40, 119991, Moscow, Russia; Lomonosov Moscow State University, Faculty of Chemistry, Leninskie gory 1, bldg. 3, 119991, Moscow, Russia

**Keywords:** L,D-transpeptidase, Mycobacterium tuberculosis, enzyme purification, recombinant enzyme, enzyme reactivation

## Abstract

*L*,*D*-transpeptidase 2 from *Mycobacterium tuberculosis *plays a key role in the formation of nonclassical 3-3 peptidoglycan cross-links in a pathogen’s cell wall making it resistant to a broad range of penicillin antibiotics. The conditions of cultivation, isolation, and purification of recombinant *L*,*D*-transpeptidase 2 from *M. tuberculosis *have been optimized in this study. Oxidation of the free SH groups of catalytic cysteine Cys354 is an important factor causing the inactivation of the enzyme, which occurs during both the expression and storage of enzyme preparations. The biochemical characteristics of purified *L*,*D*-transpeptidase 2 and *L*,*D*-transpeptidase 2 lacking domain A were determined; the kinetic constants of enzyme-catalyzed nitrocefin transformation were evaluated.

## INTRODUCTION

Tuberculosis is a dangerous infectious disease caused by *Mycobacterium tuberculosis*. The number of cases of multidrug-resistant tuberculosis is increasing with every passing year [1]. Tuberculosis therapy consists in a combined use of four major first-line drugs: rifampicin (an inhibitor of DNA-dependent RNA polymerase), isoniazid and pyrazinamide (blockers of synthesis of mycolic acids essential to cell wall formation in *M. tuberculosis*), and ethambutol (an inhibitor of arabinosyltransferase, the enzyme involved in arabinogalactan synthesis). Treatment of patients in the active phase of the disease can last up to 6 months. In some cases, *M. tuberculosis *may persist in the lungs in the so-called stationary phase, when bacterial growth slows down and neither immune response nor resistance to various antibiotics is observed [2]. In this regard, it is of great interest to search for drugs specific to the previously unknown molecular targets related to the features of vital activity and the structural organization of the causative agent of tuberculosis.

Recently, the peculiarities of cell wall formation in numerous dangerous pathogens, including *Mycobacterium tuberculosis*, have been made clear. While the penicillin-binding enzymes *D,D*-transpeptidases catalyzing the transfer of the 3^rd^ residue of meso-diaminopimelic acid (m-DAP) or L-Lys to the 4^th^*D*-Ala residue (the so called 4–3 crosslinks) play the major role in peptidoglycan crosslinking in most bacteria, most of the peptidoglycan crosslinks in *Mycobacterium tuberculosis *are formed by *L,D*-transpeptidases that catalyze transfer of the 3^rd^ m-DAP residue to the analogous residue in another peptidoglycan chain (the so called 3–3 crosslinks [3, 4], whose content in the pathogen’s cell wall can be as high as 80%).

Initially, *L,D*-transpeptidases were found in microorganisms such as *Escherichia coli*[5], *Bacillus subtilis *[6], and *Enterococcus faecium *[7]. It was not until very recently that the presence of these enzymes and their except ional role in cell wall formation in such pathogens as *M. tuberculosis* [8] and *Helicobacter pylori *[9] were revealed. This discovery cast light on the reasons for the poor efficacy of β-lactam antibiotics against tuberculosis and several other infectious diseases: unlike *D,D*-transpeptidases, *L,D *transpeptidases are not sensitive to the widely used penicillins and cephalosporins [10, 11]. The important role of these enzymes in mycobacteria functioning makes them one of the most attractive targets in the search for inhibitors that could help design novel antibiotics with anti-tuberculosis activity.

*L,D*-transpeptidases belong to the class of aminoacyl transferases [EC 2.3.2], the YkuD superfamily of proteins whose name (YkuD enzyme) originates from *B. subtilis*, the first enzyme with a known crystalline structure [6]. The *M. tuberculosis *genome encodes five proteins containing a domain with *L,D*-transpeptidase activity (Rv0116c, Rv0192, Rv0483, Rv1433, and Rv2518c regions) [11]. *L,D*-transpeptidase 2 (LdtMt2) [8] is the most actively expressed one; its presence in *M. tuberculosis *is associated with a high content of non-classical 3–3 peptidoglycan crosslinks in the pathogen’s cell wall. The amino acid sequences of *L,D*-transpeptidases from *M. tuberculosis *were identified, but their structures were established only for types 1 and 2 enzymes (LdtMt1 and LdtMt2, respectively). The LdtMt2 precursor consists of 408 amino acid residues that form the signal peptide (Met1-Ala34) and the chain of the enzyme (Cys35-Ala408), which can be divided into three domains: two non-catalytic Ig-like domains A and B (residues Ala55-Ser147 and Pro148- Gly250, respectively) and the catalytic domain C (residues Asp251-Ala408) with transpeptidase activity [8]. Cys354, His336, and Ser337, which constitute the proton transfer chain, are the key residues involved in catalysis [8]. The active center of LdtMt2 is not directly exposed to solution and is located under the so-called Tyr298-Trp324 “lid” [12] that forms three channels (A, B and C). The substrate can be delivered to the active center through two of these channels (B and C).

**Fig. 1:**
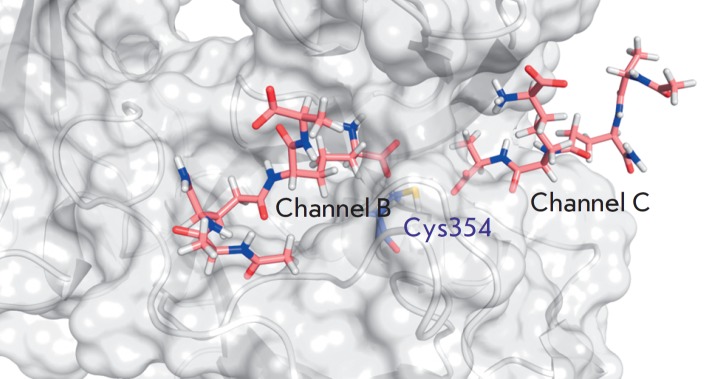
Combined frames from molecular dynamic trajectories in which peptidoglycan fragments are connected to the active site through different channels (B and C) [16]

The structure of the LdtMt2 complex with the dipeptide (N-γ-*D*-glutamyl-m-DAP) fragment of peptidoglycan in the active site of the enzyme PDB 3TUR [11] was determined. The crystal structures of the covalent LdtMt2–meropenem and LdtMt1–imipenem complexes [12, 13] were also obtained. The inactivation of LdtMt1 by various carbapenems (e.g., meropenem, imipenem, doripenem and ertapenem) was studied using the methods of pre-stationary kinetics [14]. Dhar et al. [15] demonstrated that not only carbapenems, but also faropenem (a β-lactam antibiotic belonging to the penem family) can effectively inhibit LdtMt. Our earlier molecular modeling study [16] focused on the interaction between the enzyme and the tetrapeptide fragment of cell wall peptidoglycan, as well as the known β-lactam inhibitors, and identified the features of binding of the N- and C-terminal fragments of the growing peptidoglycan chain with LdtMt2 upon formation of 3–3 crosslinks. We used these findings to build an adequate full-atom model of LdtMt2 for screening and optimization of the inhibitor structure (*Fig. 1*). A specific feature of the catalysis by *L,D*-transpeptidases is that these enzymes bind two substrate molecules at their active center. One bound molecule acts as an acyl donor that then forms the acyl-enzyme intermediate. The other bound molecule acts as a nucleophile giving rise to the 3–3 crosslink in the cell wall peptidoglycan after the nucleophile binds to acyl-enzyme and the acyl group of the L-center in the m-DAP residue of one peptidoglycan chain is transferred to the amino group of the D-center in the m-DAP residue of the other chain. Molecular modeling showed that binding of the N-terminal fragment of the growing peptidoglycan chain (an acyl donor), as well as β-lactams capable of inactivating the enzyme due to the formation of a stable acyl-enzyme, takes place in channel C, while the C-terminal (nucleophilic) fragment of the growing chain binds in channel B.

The objective of this work was to isolate, purify, and characterize LdtMt2 from *M. tuberculosis *to obtain enzyme preparations for experimental studies of the inhibitory activity of the compounds selected by computer-aided screening.

## EXPERIMENTAL

**Expression of LdtMt2 and LdtMt2_cut in *E. coli*, isolation and purification**

When conducting this study, an enzyme preparation lacking the domain A (referred to as the LdtMt2_cut preparation) was obtained, along with full-length LdtMt2. A comparative study of these two enzymes (LdtMt2 and LdtMt2_cut) could help us understand the effect of domain A on the catalytic activity of the enzyme. The pET-19b plasmid carrying either the *Rv2518c *gene (LdtMt2) that lacks the region encoding the signal peptide (Phe54-Ala408 residues) or the *Rv2518c_cut *gene (LdtMt2_cut) lacking both the signal peptide and domain A (residues Pro148-Ala408) was used for enzyme expression. In both cases, the terminal peptide consisting of 24 amino acid residues resided in the N-terminal portion of the protein: MGHHHHHHHHHHSSGHIDDDDKHM with the decahistidine terminal fragment (His-tag). *E. coli *BL21 (DE3) colonies with the transformed pET-19b plasmid were grown overnight in LB medium. Subsequently, 100 μl of the obtained culture was transferred to a flask with the LB medium containing 100 μg/ml ampicillin (Amp). The medium was incubated at 37°C and 180 rpm for 6–7 h until an optical density of 0.6–0.8 at λ = 600 nm was reached.

Enzyme expression was started by decreasing the temperature to 15°C and adding a CaCl_2_ aqueous solution (until a 2 mM concentration was reached), isopropyl-β-*D*-1-thiogalactopyranoside (IPTG) (until a 0.5 mM concentration), and glycerol (until a 2 vol.% concentration). Expression was continued for 4, 24, and 48 h. All enzyme isolation steps were performed on ice; the samples were centrifuged at 4°C. Isolation was carried out according to the standard procedure, using Protino Ni-TED 1000 columns (MACHEREY-NAGEL GmbH & Co) for purification of His-tagged proteins [17]. Cells were precipitated from the medium by centrifugation at 4,000 rpm for 15 min; the wet cell mass was weighed, re-suspended in 3 ml of LEW buffer (50 mM NaH_2_PO_4_ pH 8.0, 0.3 M NaCl); lysozyme was added until a 1 mg/ml concentration. The mixture was incubated for 30 min and then disintegrated by ultrasound in ice-cold water for 10 min. The resulting mixture was centrifuged at 12,000 rpm for 30 min at 4°C. The supernatant was collected, filtered through a 0.2 μm filter, and purified using the Protino Ni-TED 1000 column equilibrated with 2 ml of the LEW buffer. Cellular proteins were washed off by adding two portions (2 ml) of the LEW buffer. LdtMt2 was eluted from the columns with three portions (1.5 ml each) of the elution buffer (50 mM NaH_2_PO_4_, 0.3 M NaCl, 0.25 mM imidazole, pH 8.0). The total protein concentration and enzyme yield were controlled at all purification stages using the microburet method [18].

**Determination of the concentration of SH groups**

Free SH groups were titrated with Ellman’s reagent 5,5’-dithiobis(2-nitrobenzoic acid) (DTNB) using a 10 mM (4 mg/ml) solution in a denaturing buffer (0.1 M Tris-HCl pH 8.0 containing 6 M guanidine chloride). N-acetylcysteine (N-Ac-*L*-Cys) was used as a model compound to construct the calibration plot: 7 μl of the 10 mM solution of Ellman’s reagent (4 mg/ml) and 5–55 μl of a 0.2 mM N-Ac-*L*-Cys solution (2–22 μM) were added to the denaturing buffer so that the total volume of the mixture was 500 μl. The resulting mixture was then incubated for 5 min, and absorbance was measured at 412 nm. The concentration of SH groups was calculated using the extinction coefficient of the resulting 2-nitro-5-thiobenzoic acid at 412 nm and pH 8.0 (14150 M^-1^cm^-1^) [19].

**Fig. 2:**
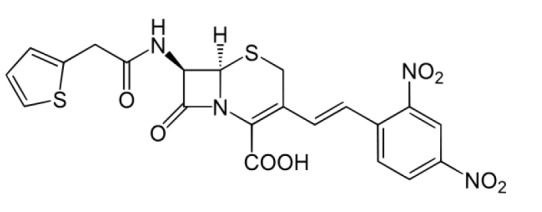
Structural formula of nitrocefin, the substrate of L,D-transpeptidase

Free SH groups in the LdtMt2 and LdtMt2_cut preparations were titrated using the same procedure: a 62–125 μl aliquot with the known enzyme concentration was added to the denaturing buffer supplemented with 7 μl of Ellman’s reagent so that the final volume of the mixture was 500 μl. The mixture was incubated for 5 min, and the absorbance at 412 nm was measured. The concentration of the SH groups in the enzyme was calculated in a similar manner as it was done for N-Ac- *L*-Cys.

**Determination of the activity of LdtMt2 and LdtMt2_cut using nitrocefin**

There currently exist no appropriate low-molecular-weight analogs of the cell wall fragment that could be used as convenient substrates for determining enzyme activity. Therefore, the activity of LdtMt2 and the reaction kinetics are studied using the chromogenic substrate, nitrocefin (*Fig. 2*) [11]. The nitrocefin-hydrolyzing activity of enzyme preparations was determined by measuring the absorption of the product of β-lactam ring hydrolysis at 486 nm in 0.02 M HEPES buffer (pH 7.5) in the presence of 0.1 M KCl. The extinction coefficient of the product was taken as 20,500 M^-1^cm^-1^ [11].

**Cultivation under reducing conditions to prevent enzyme inactivation**

Our experiments aimed at isolating and purifying LdtMt2 demonstrated that reducing conditions preventing the oxidation of the catalytic cysteine residue are needed to obtain active enzyme preparations. Therefore, dithiothreitol (DTT) was added to the medium 3 h before expression termination until a total concentration of 6 mM. Enzyme isolation was also performed in the presence of DTT.

## RESULTS AND DISCUSSION

When obtaining the LdtMt2 and LdtMt2_cut samples, we performed expression during different time periods (4, 24 and 48 hrs) to identify the conditions under which the yield of the target enzyme would be optimal. *Table 1* summarizes the results of isolation and purification of the LdtMt2 and LdtMt2_cut preparations. The optimal cultivation time was 24 h: both gain in time and higher yield of the purified enzyme were observed compared to cultivation for 48 h.

**Table 1:** Results of cultivation, isolation, and purification of LdtMt2 and LdtMt2_cut

Sample	Expression time, hrs	Raw cell mass after centrifugation, g	Protein concentration in cell extracts, mg/ml	Mass of purified enzyme, mg
LdtMt2	4	–	–	1.2
	24	0.62	3.7	2.4
	48	0.86	4.1	2.4
LdtMt2_cut	4	–	–	0.8
	24	0.71	3.1	1.9
	48	0.95	3.8	1.9

**Fig. 3:**
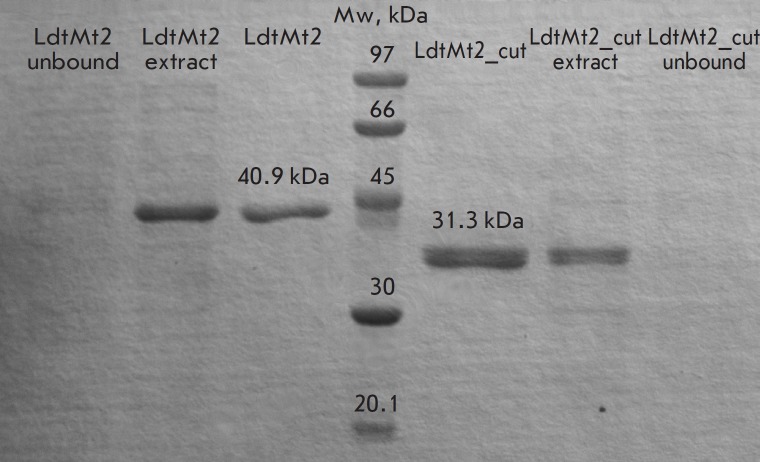
Denaturing protein electrophoresis of LdtMt2 40.9 KDa (Ala55-Ala408 + His-tag) and LdtMt2_cut 31.3 KDa (Pro148-Ala408 + His-tag) preparations purified on a Protino Ni-TED 1000 packed column, as well as cell extracts and fractions that failed to bind to the resin

PAGE demonstrated that extraneous proteins do not bind to the Ni-TED column. The resulting enzyme preparations were of high purity; their molecular weights corresponded to LdtMt2 40.9 kDa (Ala55- Ala408 + His-tag) and LdtMt2_cut 31.3 kDa (Pro148- Ala408 + His-tag) (*Fig. 3*). Meanwhile, the nitrocefin-hydrolyzing activity of the LdtMt2 preparation isolated without addition of reducing agents was significantly lower than expected. As shown earlier [11], the catalytic cysteine residue Cys354 might be oxidized, which may lead to irreversible inactivation of LdtMt2. When cultivation of *E. coli *cells and isolation of the enzyme were carried out under reducing conditions with dithiothreitol (DTT) added to the medium to prevent oxidation of the catalytic cysteine residue, the protein yield was not significantly changed and was equal to approximately 1.8 ± 0.2 mg per 50 ml of the culture medium. However, the catalytic activity of the enzyme was significantly higher. The degree of oxidation of SH groups in the LdtMt2 and LdtMt2_cut samples obtained upon cultivation and isolation of the enzyme under nonreducing and reducing conditions was evaluated by Ellman titration (see the Experimental section). The results are presented in *Table 2*. Addition of DTT to the culture medium and enzyme isolation under reducing conditions made it possible to prevent oxidation of the catalytic Cys354 and obtain a LdtMt2 preparation whose specific activity was almost twice as high.

It should be mentioned that the purified enzyme samples were stable upon storage (4°C, pH 7.5, 20 mM HEPES, 0.1 M KCl) and hardly lost their activity after a month. This fact substantially speaks in favor of the application of the obtained enzyme preparations in studying their catalytic properties and testing potential inhibitors.

**Table 2:** The proportion of free SH groups in LdtMt2 enzyme preparations obtained without and with addition of DTT to the culture medium

DTT, mM	Proportion of free SH groups [SH]/[LdtMt2], %
0	42±2
6	72±7

An important aspect of this work was understanding the effect of domain A on the catalytic activity of the enzyme. Comparison of the activities of LdtMt2 and LdtMt2_cut showed that, after domain A is removed, specific activity drops more than tenfold (see *Table 3*).

**Table 3:** Specific activity of the LdtMt2 and LdtMt2_cut preparations in the nitrocefin conversion reaction per protein content in the enzyme preparation and the content of active centers with allowance for the presence of free SH groups

Description	pdb-code (resolution, Å) (reference)	Reporting studies
aaa	aaa

**Fig. 4:**
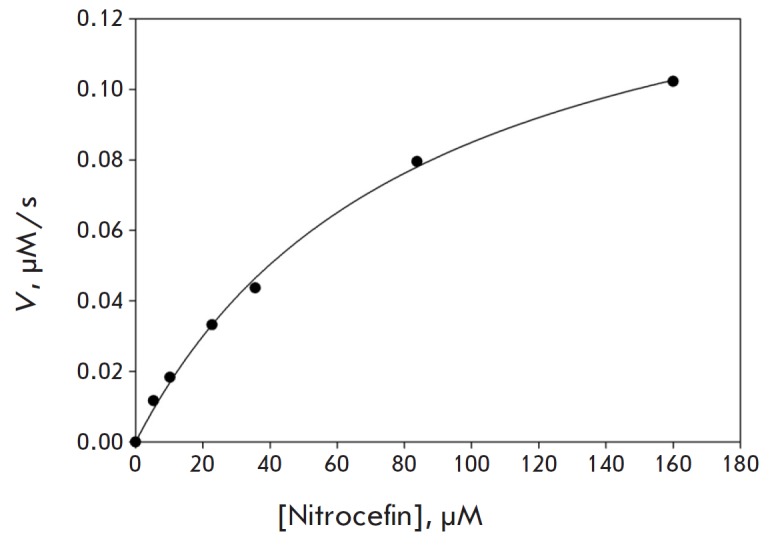
The initial reaction rate of nitrocefin β-lactam ring hydrolysis catalyzed by LdtMt2 as a function of the nitrocefin concentration. The experimental data are shown with dots. The regression curve was built using the kinetic parameters presented in the text

The K_M_ and V_max_ values of nitrocefin hydrolysis catalyzed by LdtMt2 and LdtMt2_cut were determined by analyzing the dependence of the initial rates on substrate concentrations in the range of 5–160 μM (*Fig. 4*). The concentration of the free SH groups was taken into account when determining the catalytic constants of the enzymatic reaction and evaluating the active site concentrations in the LdtMt2 and LdtMt2_cut preparations. The drop in the activity of the full-length enzyme after removal of the domain A was mainly due to the decrease in the catalytic constant of nitrocefin conversion, which went down from 0.98 ± 0.05 s-1 to 0.08 ± 0.03 s-1 when proceeding from LdtMt2 to LdtMt2_cut, while the value of the Michaelis constant worsened slightly: from 85 ± 7 to 102 ± 10 μM.

## CONCLUSIONS

Optimization of the conditions of LdtMt2 expression and purification has shown that the most productive way to obtain active enzyme preparations is to cultivate *E. coli *cells for 24 h in a LB medium in the presence of 0.2 mM IPTG, 2 mM CaCl_2_ and timely add reducing agents (DTT) to prevent the oxidation of catalytic Cys354. The resulting highly purified LdtMt2 preparation does not lose its activity after storage in 20 mM HEPES buffer, pH 7.5, at 4°C for at least a month and can be used for experimental studies of potential enzyme inhibitors selected by computer-aided screening. The biochemical and kinetic properties of a full-length LdtMt2 preparation and LdtMt2_cut preparation lacking domain A were characterized. It was shown that after domain A (which is not directly attached to the catalytic domain C) is removed, the activity of the full-length enzyme decreases significantly (more than 10 times), mainly because of the drop in the catalytic constant of nitrocefin hydrolysis. Interaction between the domains and their role in the functioning of the full-length enzyme need further investigation.

Optimization of the conditions of LdtMt2 expression and purification has shown that the most productive way to obtain active enzyme preparations is to cultivate *E. coli *cells for 24 hrs in a LB medium in the presence of 0.2 mM IPTG, 2 mM CaCl_2_ and timely add reducing agents (DTT) to prevent the oxidation of catalytic Cys354. The resulting highly purified LdtMt2 preparation does not lose its activity after storage in 20 mM HEPES buffer, pH 7.5, at 4°C for at least a month and can be used for experimental studies of potential enzyme inhibitors selected by computer-aided screening. The biochemical and kinetic properties of a full-length LdtMt2 preparation and LdtMt2_cut preparation lacking domain A were characterized. It was shown that after domain A (which is not directly attached to the catalytic domain C) is removed, the activity of the full-length enzyme decreases significantly (more than 10 times), mainly because of the drop in the catalytic constant of nitrocefin hydrolysis. Interaction between the domains and their role in the functioning of the full-length enzyme need further investigation.

## Abbreviations

Ac: acetyl
Ldt: L,D-transpeptidase
LdtMt1 and LdtMt2: L,D-transpeptidases from Mycobacterium tuberculosis type 1 and 2
m-DAP: meso-diaminopimelic acid
IG: immunoglobulin
Amp: ampicillin
IPTG: isopropyl-β-D-1-thiogalactopyranoside
LEW: lysis/equilibration/wash buffer
PAGE electrophoresis: polyacrylamide gel electrophoresis
DTT: dithiothreitol
DMSO: dimethyl sulfoxide
